# Exploring female students' perceptions of the use of digital technologies in managing academic stress

**DOI:** 10.3389/fpsyg.2023.1199038

**Published:** 2023-06-02

**Authors:** Maria-Pascale Lukenga, Laurent Billonnet, Justine Gaugue, Jennifer Denis

**Affiliations:** ^1^Department of Clinical Psychology, University of Mons, Mons, Belgium; ^2^Xlim Research Institute, University of Limoges, Limoges, France

**Keywords:** academic stress, stress management, female university students, qualitative analysis, digital health

## Abstract

**Objective:**

The purpose of this research is to explore the perceptions of female students regarding the implementation of digital technologies for academic stress management. We aim to determine if the contribution of these technologies could offer to female students a better management of the stress related to their studies and thus, a better deployment of strategies to cope with academic difficulties.

**Method:**

A qualitative study using the *focus group* methodology was conducted. Our inductive and exploratory approach allowed us to focus on the experience and perception of eleven female students from the University of Mons. The cohort was divided into two groups according to their score on the *Perceived Stress Scale-10*.

**Results:**

The data collected was analyzed using the thematic analysis of which allowed us to identify fourteen sub-themes divided into three axes: coping strategies used to manage academic stress, students' needs to improve their management of academic stress, and the implementation of technology for managing academic stress.

**Conclusion:**

Our results show that the issues present in the academic context lead students to use various coping strategies, some of which are harmful to their physical and mental health. The implementation of digital technologies and biofeedback seems to be an approach that could help students adopt more functional coping strategies and alleviate their daily difficulties in managing academic stress.

## 1. Introduction

In the academic environment, students are challenged with many responsibilities and high achievement requirements that demand constant adaptation on their part. This causes great upheaval in their lives as students and is an intense stressful experience (Véron et al., 2020). The concept of stress by Lazarus and Folkman (1984) provides an understanding of the mechanisms underlying the emergence of academic stress in the student population. For example, students feel stressed when they feel that they lack the skills necessary to meet the demands of the academic environment (Barker et al., 2018). Yet, stress can be beneficial for this population because it increases the motivation of students in the face of the various demands of the university environment (Ganesan et al., 2018). However, when the presence of this stress comes to disrupt their learning or coping skills, it becomes detrimental to their health and studies (Deasy et al., 2014). An international study on student stress conducted in nineteen universities in eight different countries, including Belgium, observes the presence of psychological pathologies in university students, of which the most frequent are depression (21.2%) and anxiety (18.6%) (Auerbach et al., 2018). Other studies report the presence of eating disorders, sleep disorders, excessive alcohol and/or substance use that result from this chronic exposure of students to academic stress (Boujut et al., 2009; Dahlin et al., 2011; Morvan et al., 2016; Romo et al., 2019). In consideration of these different manifestations, the student population proves to be an at-risk population, and effort is required to minimize the negative effects of stress on the physical and mental health of students.

### 1.1. Student resources for academic stress

Different *coping* strategies are spontaneously implemented by students to deal with difficulties related to the academic environment (Lassarre et al., 2003; Freire et al., 2016). These coping strategies can be effective (e.g., positive reinterpretation or seeking social support) or ineffective (e.g., alcohol consumption or self-blame) for reducing their academic stress (Spitz et al., 2007; Lefèvre et al., 2020). However, it has been found that students use more *coping* strategies in the academic context that prove to be non-effective, which is not without consequences for their physical and mental health (Deasy et al., 2014; Morvan et al., 2019). To remedy this, methods based on cognitive or behavioral approaches or mindfulness (such as *Mindfulness-based stress reduction* MBSR) have been able to demonstrate effectiveness in managing academic stress and can be offered to students (Regehr et al., 2013; Berrewaerts and Desseilles, 2016). In addition, at some universities, students can also benefit from interventions based on stress reduction methods through psychoeducation workshops, stress management training, and support groups (Worsley et al., 2022). In addition to these types of offerings, some universities provide more personalized interventions for their students through facilities where they can consult with a health professional (Frajerman, 2020). However, research shows that students very rarely use the services offered to them because, for some, they feel prejudiced about their mental difficulties (Atik and Yalçin, 2011) or, for others, because of the inaccessibility (e.g., price, waiting time) of these services (Berrewaerts and Desseilles, 2016; Anderson et al., 2017). As a result, they are left without the ability to manage their stress levels and could experience persistent psychological or physical symptoms, which has led researchers to examine alternative stress management methods to help students effectively use their coping strategies (Regehr et al., 2013; Berrewaerts and Desseilles, 2016; Servant et al., 2016).

### 1.2. Integrating technology into stress management

To address barriers to seeking help, many stress management programs are made available online on the Internet in the form of self-help (Day et al., 2013). These programs, based on cognitive-behavioral approaches, offer an alternative to therapist-facing psychological treatment (Cuijpers et al., 2010). In addition, they have the advantage of being accessible to a large number of people by avoiding stigmatization because of their anonymous nature (Davies et al., 2014). They also allow the individual to be an actor in their health and to benefit from greater independence in their care process, which, in the long run, can improve self-care skills (Golchert et al., 2019). However, for some authors, effective management of stress levels through these self-help programs is difficult due to the subjectivity of their assessment of stress levels through self-report psychometric questionnaires (Servant et al., 2016; Pourmohammadi and Maleki, 2020; Parlak, 2021). This is because individuals are generally unaware of their stress state and social desirability biases may occur, thus altering the written data (Arza et al., 2019; Kang and Chai, 2022). As a result, stress reduction interventions may be inappropriate for the individual's actual state, as they are derived from non-objective outcomes (De Witte et al., 2019).

The technology aims to overcome certain measurement limitations of the tools usually used in stress management and it offers individuals a precision of measurement that makes it possible to provide stress management programs more suited to their condition (De Witte et al., 2019; Gedam and Paul, 2021). Devices using biofeedback in stress management, for example, increase the autonomy of individuals, by providing them with the ability to self-regulate their physiological indices in real time (Kennedy and Parker, 2019). Through the use of sensors, these tools measure various physiological parameters which indicate the presence of stress, such as blood pressure, skin temperature, electrodermal activity, and respiration. The parameters can be measured by electrocardiogram (ECG^1^), electroencephalogram (EEG^2^), photoplethysmogram (PPG^3^) or electromyogram (EMG^4^). There are also other sensors that can indicate the presence of stress in the body through the measurement of the level of certain hormones, such as cortisol (Yu et al., 2018a; De Witte et al., 2019; Gedam and Paul, 2021; Hickey et al., 2021). Moreover, many technologies which are available to the general public (connected watches, fitness trackers or smart home health devices) can also allow an individual to monitor their stress in real time through their physiological data (Sykianaki et al., 2019; Samson and Koh, 2020; Hickey et al., 2021). However, these data are only the result of a stress state; it is necessary that they can be interpreted in a user-defined context or that the user is psychologically assisted in the decryption of these constants. Furthermore, these devices do not provide access to tools that offer the possibility of regulating stress levels. Some applications available on smartphones can compensate for this deficit. Indeed, they offer the possibility to access stress management programs including well-proven methods (e.g., mindfulness, breathing techniques or meditation). They aim to improve the coping strategies of users but are not developed to accurately assess the state of stress (Samson and Koh, 2020). Moreover, the psychological factor, which includes the psychological mechanisms underlying the functioning of an individual, is also often forgotten as an input parameter of these technologies, despite the fact that it is important and not negligible in stress management (Schlatter et al., 2021; Umair et al., 2021).

## 2. Present study

In view of all these statements, it is now essential to provide students with new ways to help them to reduce their stress levels and thus decrease the risk of developing somatic or mental pathologies. We have suggested before that the evolution of technology could allow more accurate methods of measuring stress to be proposed, leading to the possibility of identifying the factors that cause this stress and to acting on them to reduce it. In addition, it allows the user to manage their stress more easily and independently by using the biofeedback method. It might be appropriate to provide the students with technological assistance that would aim to inform them of their stress state in real time and at giving them targeted tools to regulate it. Yet this kind of assistance must be fully accepted by this population. Also, there seem to be a limited number of studies in the francophone research community that have applied this type of approach with students (Saleh et al., 2018; Romo et al., 2019), which reinforces our desire to make progress on this topic.

### 2.1. Study aims

The potential of technological innovations in this area led us to examine more closely the perceptions of university students on the use of this technology in the management of academic stress based on their subjective experience of this stress. Our research question is ***how do university students perceive the use of digital technologies in managing their*** ***academic stress****?*

To answer this research question, we developed three sub-questions:

- What resources do students typically use to reduce their stress conditions?
- What are their current needs to reduce their academic stress?
- How do students consider the use of digital technologies in managing their academic stress?

## 3. Materials and methods

### 3.1. Setting

The qualitative method of focus group (FG) was chosen to collect the subjective experiences of our group of female students. This inductive and exploratory method focuses on each participant's experience and perceptions regarding the proposed topic. The FG also offers the possibility of investigating the needs and expectations of the target population to develop a better understanding of the research problem (Moreau et al., 2004). In addition, this research is part of the continuity of the U-Stress project that has been led by Professor Denis at the University of Mons (UMONS) since 2020. It aims to establish an inventory of the experience and management of stress at the University. The research protocol of this study was approved by the Ethical Committee of the University of Mons before being submitted to the participants.

Our study was conducted among female UMONS students in Belgium during the 2021–2022 academic year. It should be noted that during this period, health restrictions due to the 2019 coronavirus (COVID-19) crisis were in effect in Belgium, such as wearing masks, limiting social contact, and both distance and face-to-face teaching for higher education students (Service public fédéral Santé publique, 2021, 2022).

### 3.2. Participants and recruitment

Our sample consisted of 11 female students, aged between 18 and 22 (*m* = 19.64, σ =11), divided into two separate groups based on their score on the Perceived Stress Scale 10 (PSS 10) by Cohen et al. (1983). Our participants were recruited via the social network Facebook and directly on the UMONS campus. The students were informed of the research objectives and the practical modalities of participation in these FGs via an information letter. The inclusion criteria were that the students should be enrolled and studying at UMONS, regardless of their year or field of study. These students were required to be aged between 18 and 25, which corresponds to the average age of students enrolled at universities in Belgium.^5^ It was also necessary that the research participants could understand and communicate fluently in French. Our exclusively female recruitment is not surprising considering that in the scientific literature, women generally obtain higher levels of self-reported stress and physiological responses than men (Yikealo et al., 2018). Therefore, they may have been more interested than men in participating in our study.

### 3.3. Description of the participants

The qualitative FG method used limited the recruitment of participants to a minimum of four people to ensure a group dynamic and a maximum of twelve to allow for each candidate's expression. Typically, the focus groups were composed of five to eight people. Thus, based on this range, we developed two focus groups in which an average of six candidates per group were divided based on their stress levels on the PSS-10 (Krueger and Casey, 2014). Furthermore, in the context of qualitative research design, the importance of the study does not depend on the number of participants. In contrast to quantitative studies that focus on statistical power and generalizability, qualitative research aims to gain a comprehensive understanding and provide detailed descriptions of a phenomenon (Vasileiou et al., 2018). Therefore, sample size determination in qualitative research is often guided by the principle of data saturation. This principle suggests that data collection should cease when participants no longer provide new information or insights (Saunders et al., 2018). Thus, the value of a qualitative study lies in the depth and richness of the data obtained, rather than in the number of participants, which is relevant to the research goals and objectives of our qualitative study (Vasileiou et al., 2018).

Students in both groups were free of psychiatric disorders, and none of them was taking any medication that would potentially affect mood, nor did they receive any psychological follow-up. A more detailed description of the characteristics of these participants based on their assigned group is presented in Table 1.

**Table 1:** Socio-demographic characteristics of the students according to group distribution.

	**Group 1 (*N* = 5)**	**Group 2 (*N* = 6)**
**Age (year)**		
Average (SD)	19.8 (1.17)	19.5 (0.76)
Scope	19–22	18–20
**Scholarship status** ***n*** **(%)**		
Yes	1 (20%)	4 (66.7%)
No	4 (80%)	2 (33.3%)
**Student job during school year** ***n*** **(%)**		
Yes	–	2 (33.3%)
No	5 (100%)	4 (66.7%)
**Home** ***n*** **(%)**		
Student housing	1 (20%)	1 (16.7%)
Family	4 (80%)	5 (83.3%)
**Faculty** ***n*** **(%)**		
Faculty of Medicine and Pharmacy (FMP)	4 (80%)	–
Faculty of Translation and Interpretation (FTI)	1 (20%)	1 (16.7%)
Faculty of Psychology and Educational Sciences (FPES)	–	4 (66.6%)
Faculty of Science (FS)	–	1 (16.7%)
**Year of study**		
Bac 1	–	2 (33.3%)
Bac 2	5 (100%)	–
Bac 3	–	4 (66.7%)
**Mean score on the PSS-10**	27.2	17

### 3.4. Measures

#### 3.4.1. PSS-10

In order to design the focus groups according to the perceived stress levels of the female students, the Perceived Stress Scale (PSS-10) was provided. The PSS-10 (Cohen et al., 1983) is a ten-item questionnaire that assesses stress levels in individuals aged 12 years and older. The French version used was validated by Bellinghausen et al. (2009). The PSS-10 measures the degree to which an individual perceives life as unpredictable, uncontrollable, and overwhelming. This questionnaire enables respondents to rate their feelings and thoughts during the past 30 days on a five-point Likert scale (from 1 = never to 5 = very often). Individual scores on the PSS-10 can range from 0 to 40 and are categorized into three groups: 0–13 corresponds to low perceived stress, 14–26 corresponds to moderate perceived stress, and 27–40 corresponds to high perceived stress.

With the absence of a validated and French-translated scale on the perception of academic stress for university students in the scientific literature, the use of the PSS-10 provided an overview of the general level of stress among these students. The results of the PSS-10 were then put into perspective with the students' discourse in order to better understand their experience of academic stress.

### 3.5. Procedure

#### 3.5.1. Focus group

By conducting an FG, the researcher was able to interview several participants simultaneously, so agreements and disagreements could emerge among them. Thus, the participants were able to debate, in a constructive manner, around the proposed theme and bring additional information to the study. The questions that we developed could be confronted with the reality of this population and also be adapted according to the ideas that emerged from the discussions (Moreau et al., 2004). In developing the questions, we considered the relevant literature on this topic, as well as established scientific techniques for developing interview questions. Specifically, we reviewed studies on similar topics and conducted an in-depth analysis of the research questions and interview protocols used in those studies. We also consulted with experts in the field, such as university professors, to ensure that the questions were appropriate and complete. These techniques allowed us to develop questions that effectively captured relevant information while maintaining a consistent structure among interviews.

#### 3.5.2. Conduct of the FG

Participants were contacted by email to participate in the FG sessions following their online completion of the PSS-10. The time between the completion of the PSS-10 and the participation in the FG varied for each candidate. Two separate FG sessions were conducted with each of the two groups by a moderator and an observer who were a Master 2 student in Clinical Psychology, the author of the article, and a Professor in Clinical Psychology. The FG sessions took place in a neutral and quiet meeting room at UMONS. The choice of this location provided an anonymous setting for the research and facilitated the flow of the discussions (Moreau et al., 2004). During these sessions, the students were asked to answer to the questions of the pre-established interview guide, which helped to ensure fluid communication, without being limited to too rigid an interview structure (Leclerc et al., 2011). This attitude allowed a reflexive and exploratory view to be maintained that allowed us to not unduly guide the interviews according to the interests or preconceptions of our research (Yelnik, 2005). In order to have a better grasp of their subjective experience of dealing with academic stress, the presence of an observer was also necessary to take note of the students' turns of speech and non-verbal attitudes during the FGs (Touboul, 2012). To facilitate this notetaking, students gave their consent to have their names placed in front of them. By the end of the second interviews, theoretical saturation was reached, telling us that no more new information could come from the interviews (Krueger and Casey, 2014). As a result, the FGs were reduced to two sessions, averaging 1 h and 40 min in length. Only one of the five participants in the first group did not participate in the second focus group session. In the second group, all participants attended the entire focus group. After the participants signed a consent form, all sessions were voice-recorded to allow the researcher to fully concentrate on the participants' speech and on the responses. Moreover, this limited the loss of data and facilitated the verbatim transcription necessary for an accurate analysis of their speech.

#### 3.5.3. Data analysis

Descriptive statistics (mean and standard deviation) were used to describe the characteristics of the sample and the scores of the participants on the PSS-10. To explore potential differences between groups based on their PSS-10 scores, an inferential statistical test, specifically an independent samples *t*-test, was performed using R software. The qualitative data obtained from the focus groups were analyzed using the thematic analysis of Braun and Clarke (2006) via the qualitative data analysis software Nvivo 12. This analysis, which had to be systematic, sequential, verifiable and continuous, allowed us to identify and analyze the themes that emerged during these discussions. Thematic analysis is not a linear approach; it is a dynamic process that led the researcher to go back and forth between the six different steps established by the authors. This method led to the exploration of the implicit and explicit meanings of the information collected during the focus groups. Thus, we had to go beyond the superficial meanings of the data in order to give them meaning.

## 4. Results

### 4.1. PSS-10

The sample was divided into two groups based on scores on Cohen et al.'s (1983) Perceived Stress Scale-10 (PSS-10).

The first group (see Table 1) was composed of students with high PSS-10 scores (*m* = 27.2, σ = 2.49) compared to the total sample (*m* = 21.63, σ = 6.76). The second group (see Table 1), on the other hand, was elaborated according to the low score of the students on the PSS-10 scale (*m* = 17, σ =5.44) compared to the total sample (*m* = 21.63, σ = 6.76).

To determine if there was a significant difference between the two groups, we performed an independent samples *t*-test. The results indicated that there was a significant difference in PSS-10 scores between group 1 and group 2, *t*_(9)_ = 3.174, *p* < 0.05, Cohen's d = 2.06. Specifically, PSS-10 scores in group 1 were significantly higher than those in group 2.

Overall, our results suggest that individuals in Group 1 experienced higher levels of perceived stress compared to individuals in Group 2.

Thus, in our sample subdivided into two groups, we can observe in the high stress level group, 80% of these students were from the medical field. This is in contrast to the second group, in which all the candidates were from a non-medical field. In addition, we note that the students with high levels of stress were also predominantly from the second year of the bachelor's degree program (see Table 2). Those with the lowest levels of stress were predominantly from the end of the bachelor's degree program.

**Table 2:** Description of students by faculty and year of study and PSS-10 score.

**Participants**	**Faculty**	**Year of study**	**PSS-10 score**
**Group 1**			
E1	FMP	Bac 2	28
E2	FMP	Bac 2	29
E3	FTI-EII	Bac 2	27
E4	FMP	Bac 2	23
E5	FMP	Bac 2	29
**Group 2**			
E6	FPSE	Bac 3	15
E7	FPSE	Bac 3	20
E8	FPSE	Bac 3	7
E9	FPSE	Bac 3	22
E10	FS	Bac 1	20
E11	FTI-EII	Bac 1	18

### 4.2. FG analysis

The data analysis revealed 14 sub-themes (see Table 3) related to the subjective experience of these female UMONS students in managing their academic stress. These sub-themes were divided into three distinct thematic areas which are: coping strategies used to deal with academic stress, students' needs to improve their management of academic stress, and the implementation of technology as a tool in managing academic stress.

**Table 3:** Organization of the established themes emerging from the subjective experience of female students from the University of Mons on the management of academic stress.

**Thematic axes**	**Coping strategies used to deal with academic stress**	**Students' needs to improve their academic stress management**	**Implementation of technology as a tool in the management of academic stress**
Themes	•Making the right choice in studies •Letting things go •Limiting the over-investment in studying •Focusing more on my wellbeing •Social support is important! •Why not a mental health professional?	•Inundated with information, how to better transmit it? •Learning to manage my workload •Better listen to the warning signs that come from my body •I resist therefore I am! At what cost?	•Biofeedback as an aid in detecting stress •(Good) communication above all •Digital support between students is a great way to help each other •It's not only about technology

#### 4.2.1. Coping strategies used to deal with academic stress

##### 4.2.1.1. The right choice of studies

The participants first spoke of their complicated transition from high school to university. The female students were confronted with a new academic environment in which they had to learn to deal with many unknowns and doubts, including whether they had made the right choice of studies, which they felt was a major stress factor.

“*The beginning of university is quite complicated because you come out of the high school and it's something different and you don't know what to expect. You don't know if you've made the right choice of studies. You're not sure yet if you're going to like it or not. And then there's the whole organization thing. The course materials are very different. So that stressed me out at first” (E4)*.

We effectively observed a lower perception of academic stress, and lower scores on the Perceived Stress Scale (PSS-10), in the participants who are confident that they have made a good choice of studies and working methods than in the other female students.

“*I don't feel like I stress that much. I just chose a subject that I knew I would be good at” (E8)*.

Furthermore, when they objectively observed an improvement of their academic performance as a result of their efforts to adapt to university studies, their self-confidence increased leading to the decrease of their academic stress.

“*Stress? It's much better than last year. I found my way of working, and I saw that I passed my exams, so I felt that I was competent” (E10)*.

##### 4.2.1.2. Letting things go

Confronted with the various issues present in the university environment, some female participants expressed how difficult it can be for them to experience failure. By taking a step back from the challenges of success, some students were able to transform this subjective experience, initially considered negative, into a meaningful and enriching one.

“*Well, I also put things into perspective, in fact. When I fail, it makes me want to do better. So, I don't know if this will reduce stress, but I tell myself that I had the ability to do it, even if sometimes I can't see it. I mean, I don't see it. I try to move forward like that. [...]” (E9)*.

Rationalizing also allows students to let go when academic stress becomes high, which they explain is an important protective factor against the various consequences that stress can have on their health.

##### 4.2.1.3. Limiting the over-investment in studying

For our female participants, and particular for those from the medical field, the university experience can be marked by high pressure to succeed, which can come from others, but especially from themselves. The desire to succeed goes beyond academic or even family requirements, which puts a lot of pressure on these female students.

“*I will study TOO much and do useless stuff just to make myself feel good. I have my exercises and I'll do absolutely all of my exercises again, even if I did them right the first few times, just to be sure that on the exam it will be okay” (E10)*.

This strategy, which can give the students an illusion of control over the situation, can prove to be a failure and, in addition to the stress, bring psychological suffering.

“*On the other hand, classes really stress me out. If I don't work some days, I feel guilty. As a result, I don't feel very well, which leads me to work harder the next day. It still stresses me out quite a bit” (E6)*.

##### 4.2.1.4. Focusing more on my wellbeing

In the face of significant stress, some of the participants can cope with stress by engaging in leisure activities (such as walking). These activities help to bring about a state of wellness in them, which contributes to a decrease in their perceived stress levels.

“*When I'm stressed, I try to go outside, take a walk, clear my head, and then I feel much better” (E11)*.

Others, in contrast, make use of technological tools, such as smartphone relaxation apps or online hypnosis videos, to lead to this state of wellness. However, the practice of these “relaxing” methods is not suitable for everyone. These techniques require the students to know when to let things go, which is difficult to achieve due to lack of time and training.

“*I had tried another application with some free meditations or breathing exercises. But I realized that it gave me panic attacks, so I stopped” (E3)*.

##### 4.2.1.5. Social support is important!

The social support that these students receive from their families and/or friends is their main resource for dealing with academic stress. Indeed, it seems that the social support they receive from their families and/or friends allows them to temporarily distance themselves from the source of their stress, which is their studies, and this is an important protective factor for our participants against academic stress.

“*My parents think that I study too much. They are more likely to take my notes so that I don't get too stressed out” (E5)*.

However, the female students sometimes have difficulties to solicit their social circle because of the fear of disturbing the other, thus leading our students to keeping their adversity to themselves.

“*I find it difficult to express myself when things are not going well. In fact, I prefer to keep it to myself so that I don't bother others. Just the fact of disturbing others, I am uncomfortable in fact. So, I don't necessarily go to others” (E4)*.

The participants also emphasized the support and mutual help between peers that helped them in their academic career and sometimes directly in the management of the stress that comes from the difficulties encountered in this career. Indeed, the fact that they share the same academic environment as their peers allows them to obtain advice and emotional support that is more adapted to their academic reality.

“*Yes, there is a lot of peer support. A lot of people sharing their experiences, whether it's from the years above or the same year, etc. A lot of sharing of notes and it's true that it helps a lot. And moral support, lots of little words of encouragement, kindness, etc.” (E2)*.

##### 4.2.1.6. Why not a mental health professional?

When the difficulties are too much and/or when the social support and/or other resources in place are no longer sufficient, some of our female students seek help from a psychologist, which they consider to be more appropriate for their situation. Having a person whose “job” it is to listen also allows them to speak more freely about the difficulties they are going through.

“*I'm not someone who talks a lot because I don't like to bother people. But I tell myself that if I have a specialist in front of me, well, that's why they are there. So, I will talk longer, or at least I will explain more things to them” (E8)*.

However, a strong feeling of stigmatization is present among some of our female students concerning the use of psychologists available on the UMONS campus. Preconceptions about these services are expressed in the discourse of the female students. According to them, the use of these services is only for students with severe mental health problems.

The necessity of distancing themselves from the academic context in order to talk more freely about their stress management difficulties was also mentioned. Other female students, on the other hand, emphasized the necessity of having this type of service on campus because it increases the accessibility of mental health care. For those students who did use these services, they did not get the help they were looking for. As a result, the process of seeking care began, but was quickly abandoned by the female students, leaving them with no help adapted to their situation.

#### 4.2.2. Students' needs to improve their academic stress management

Through this thematic axis, we have listed the needs of these students in order to have some ideas about how to better manage their academic stress and mental health.

##### 4.2.2.1. Inundated with information, how to better transmit it?

The difficulty of obtaining information from the university related to their academic studies in a correct manner is a very important part of students' academic stress and this problem was mentioned by all the students. The first sub-theme is the lack of advanced information regarding the planning of learning activities, and the failure of communication, which affects the students' organization. The students in our groups report that this lack of anticipation by the university regarding the announcement of important information (e.g., exam dates) impacts their work time management, which leads to difficulties in managing the learning situation, thereby increasing their academic stress.

“*We get a lot of last-minute information and I need to plan things well in advance. [...] It stresses me out a lot. I need a lot of organization and UMONS doesn't provide it” (E2)*.

The second sub-theme, closely related to the first, addresses the need for a better form of communication from the University of Mons. The length and frequency of the emails sent by the university are, according to them, not ideal to transmit information.

“*[...] even when we see the emails, they are much too long. And with the services, and especially the administration, we must communicate a lot with them, for our annual student program. And it's really a disaster [...]” (E10)*.

Thus, to obtain information related to their university life, they seek information from the different university support groups present on social networks (e.g., Facebook and Instagram) that they use every day. The female students are asking the university to communicate in a similar way to social networks, in short, simplified and instantaneous messages. However, this goes against the norms and expectations of the university system, and those of the professional world that they will experience after their studies, which require a longer and more complex communication of information, requiring more effort of adaptation from the students.

##### 4.2.2.2. Learning to manage the workload

The amount of work required to complete their academic year in a very limited amount of time is one of the difficulties largely encountered by our female participants. This amount of work is such that they say they feel they no longer have time for themselves, for their loved ones, or for the activities necessary for their wellbeing.

“*I feel a little stressed, we have too much practical work. And I feel like I don't have time for myself, for my family, to do activities, especially in the last two years” (E7)*.

It is also difficult for them to allow time to see a psychologist when they are experiencing significant difficulties in their academic course. For some female students, the idea of taking this time brings more stress because it would be to the detriment of work time.

“*Right now, I don't have too much time to go, since I have to study, and I have to dedicate my time to studying. Right now, I know well, I would be more stressed to take that time, if it's to go see the psychologist, than to keep the time to study. Especially during this period” (E1)*.

Managing workload and class schedules is a challenge. While for some, the use of mobile time management applications can solve the problem, others hope that the university can help them to solve it through workshops on work time management that are adapted to the reality and specificity of their academic program.

##### 4.2.2.3. Listen better to the warning signs that come from my body

For our participants, the presence of academic stress is often only perceived when the various clinical signs of stress appear. For some of our participants, this stress, when it occurs, can lead to nervous tics that can sometimes cause infections and scarification.

“*Me too, I rip the skin off my lips and the little bits of skin around my fingers” (E3)*.

“*Uh me too. With my stress, that's something I do; scarification” (E5)*.

The manifestations of stress also influence their academic performance.

“*I wanted to add that when I study and I'm stressed, I find that I'm slower and that I slowdown in the work that I would have done without the stress. So, stress has a big impact on my studies” (E11)*.

##### 4.2.2.4. I resist, therefore I am! At what cost?

During these focus group sessions, the need to not change anything emerged strongly from several participants. For them, stress is necessary in certain situations and there is a fear of the different possibilities that would allow them to improve their situation. This leads to a great resistance to change on their part.

“*I found out how to study and everything. I mean, I don't want to go somewhere where they're going to stress me out more than anything else by giving me other methods and telling me: oh my God, it's my method that's not good and all. Whereas, for the moment it's fine” (E10)*.

Also, these participants expressed that the need to change their working methods or stress management in order to adapt to academic requirements was no longer relevant. For some of them, the need to improve their conditions in the face of academic difficulties was present at the beginning of the course. Finally, they succeeded in finding the resources to face these challenges on their own.

#### 4.2.3. The implementation of technology as a tool for managing academic stress

To illustrate a technological assistance device that could be used in the management of academic stress, we provided students with an example of a portable device that uses a combined approach of physiological and psychological stress measurement. Thus, the students would be led to better understand their physiological reactions related to stress in the context that they had indicated in order to distinguish the situations that contribute to their academic stress.

##### 4.2.3.1. Biofeedback as an aid in detecting stress

The students expressed nuanced opinions on the usefulness of a technological device aimed at detecting and reducing their academic stress in their daily lives and which could indeed help them prevent the accumulation of stress and thus limit its physical and/or psychological manifestations. One concern is that such a device would search for all types of stress or would accentuate innocuous stress events, thus leading to more stress.

“*I think, if I have a device that tells me I'm stressed, well that's going to stress me out even more. So, I don't think it's a good idea. Because if you don't know it, after a while it will pass by itself, you think about something else. But if we tell you: yes, you're stressing, you'll just think about it” (E6)*.

In the end, the students agreed on the relevance of having wearable devices that can instantly identify their stress levels and then help them to temporarily manage their stress through stress reduction programs.

They also stated that it would be preferable for these portable devices to be linked to fixed devices in the home, because they emphasize the importance of the atmosphere in which we are in order to manage stress effectively. Therefore, the management of this stress could be pursued at home with the help of lighting or relaxing smells that could create an environment that would reduce stress.

“*I think it could also be interesting to have something permanent. For example, relaxing smells, or even lights, I really think that can be helpful. I think that the atmosphere in which we put ourselves influences our state a lot” (E1)*.

However, students' motivation to use such devices remains a barrier.

##### 4.2.3.2. (Good) communication above all!

The difficulties of communication and organization from the university, emphasized by our participants, show a need for them to find information more easily and that this information has to be clear and concise. The female students suggested that the presence and the accessibility of the information should be strengthened by using social networks to reach this goal. Our students would like to have communication that is close to their culture and their tastes to reduce both their mental load and their difficulties to adapt to the university communication.

“*[...] on Facebook, there is less blah [...] we don't get the emails from Moodle just because the teacher has put a question, etc. It's more visible on Facebook, so maybe it's better to go through those channels” (E5)*.

The need for these female participants to have centralized information in a single place, as well as instructions on how to deal with the different academic requirements, was also observed during the focus group sessions.

This would help the students to obtain the information necessary for their academic course and reduce the stress associated with it.

#### 4.2.4. Digital support between students is a great way to help each other

Despite some of the mentioned disadvantages (e.g., bad people), the majority of the female participants were in favor of the proposal to create a digital student self-help community. However, for some of our participants, this mutual support must be limited to the academic sphere, and for others, to the administrative sphere. Some students expressed that they are not interested in receiving help in the academic sphere from other students because they have found a balance in their academic career on their own. Others were concerned that other students would provide support without respecting their specific student reality, which would cause a new form of academic pressure for our students.

“*Because I feel like, I can handle the other stuff. [...] If I go, it's going to stress me out more than anything else to say to myself, there are other things to do or other potential things that could work too. But for me, if it works, I don't want to look elsewhere” (E6)*.

Then, regarding the sharing of their personal difficulties related to the academic context, the fear of judgment from others, as well as the confidentiality of their sharing, were considered important points of concern for our students. However, they also mentioned that the anonymous nature of the relationship with the other person could be a barrier to the creation of a trusting relationship and thus a barrier to self-disclosure.

“*I think I need to really trust the person to tell them anything about myself. And no, I don't think with people, even if they're going to confide in them too and everything, I don't think I'll do that” (E10)*.

##### 4.2.4.1. It's not only about technology

The participants also emphasized the benefits that talking and/or listening spaces could bring, without explicitly indicating the need for a new technological tool.

A first sub-theme concerning the talking spaces came from the fact that, during these FGs, the students were able to express their difficulties and benefit from peer support that led them to reconsider some situations and not feel alone anymore. Therefore, for these students, having group or individual places to talk could be beneficial in managing academic stress.

“*Personally, it reassured me a little because I realized that I wasn't the only one, because they think a little like me. It reassured me” (E9)*.

The second sub-theme concerns the listening support services, such as the telephone line which is provided when other services are unavailable and the need to talk is urgent. However, for some participants, the need for face-to-face contact in these difficult situations is crucial, which makes this resource inappropriate for them.

## 5. Discussion

Our goal was to explore how female students perceive the use of digital technology and biofeedback as tools that can help them improve their academic stress management.

### 5.1. Coping strategies used by the female students in the academic context

**Figure 1:**
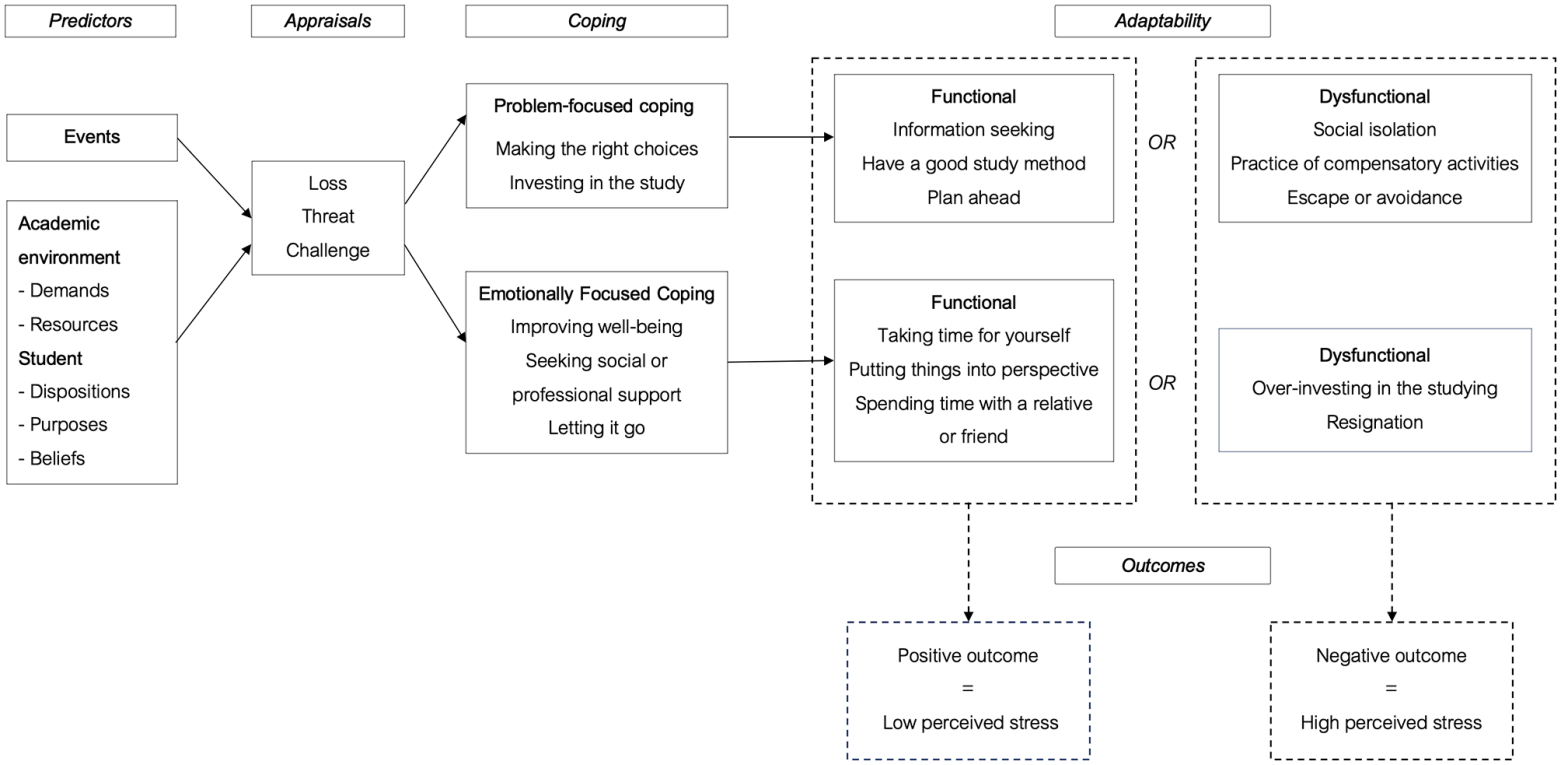
Scheme of the coping strategies used by the female students at the University of Mons based on the Lazarus and Folkman (1984) model of stress and coping developed by our research team.

To answer to our research question, we investigated the coping strategies that these students use to deal with their academic stress. Through Lazarus and Folkman's (1984) theory of stress and coping, we know that cognitive appraisal and coping are the main mediators of the transactional relationship between people and their environment. Thus, faced with a stressful situation, our female students evaluate the resources they have to cope with the requirements of the academic environment. Then, following this subjective evaluation of the demands and resources, the students orient their responses to the stress according to emotion-centered or problem-centered coping strategies. At the end of our analyses, we listed (see Figure 1) some of the coping strategies used by our students to visualize those that may be relevant or harmful in the management of academic stress and may influence its perception. It is important to emphasize that there is no strategy that is effective or ineffective. However, the use of problem-focused coping strategies is more appropriate for dealing with academic stress in the long term. In contrast, emotion-focused coping strategies are only effective in the short term or when the event is uncontrollable (Bruchon-Schweitzer, 2001; Lassarre et al., 2003).

Furthermore, we observe in our results that the perceived stress of female students from medical fields was higher than that of female students from non-medical fields. This finding, regularly observed in the literature, could result from a higher perception of the demands of success in the medical field which leads these female students to exhibit higher levels of stress than their peers from other fields of study (Moreira de Sousa et al., 2018; Romo et al., 2019; Seedhom et al., 2019).

Our analysis also revealed that the perceptions of stress among female students tended to decrease as their academic years progressed. As a result of the various coping strategies deployed to adapt to the demands of the academic environment, the participants were able to decrease their feelings of stress over their year of study (Wyatt and Oswalt, 2013). However, despite their efforts, the majority of our female students still have high scores on the PSS-10, indicating that stress persists in their daily lives. Moreover, these students, with high levels of stress, are more likely to use coping strategies focused on emotion (Teixeira et al., 2021) which in the long term are not very effective and are harmful to their health (Parada and Verlhiac, 2022), and which can lead to the persistence of psychosomatic manifestations of stress in them. Therefore, in order to reduce the high perception of this stress, it is preferable to get students to use coping strategies focused on the problem (Montero and Morales-Rodríguez, 2021). Mazé and Verlhiac (2013) already highlighted the possibility of acting on coping strategies by promoting student support and coaching devices within their institution. This would allow students to find adequate coping strategies according to their resources and the academic environment as well as prevent the onset of stress symptomatology (Karaman et al., 2019).

Secondly, we can observe in all our participants that perceived social support has a beneficial and important role in the feeling of stress and academic performance (Poots and Cassidy, 2020). However, this support, which may come from family members, friends, or peers, remains difficult to mobilize for some of our students, for fear of bothering the other. This contributes to maintaining their high levels of stress. In fact, people with high levels of perceived stress are not always able to use their social support optimally (Ioannou et al., 2019). Thus, in order not to leave them struggling, other social supports must be offered to them, such as the use of a health care professional. However, despite the importance of this support, it remains stigmatized or considered inaccessible by many people including some of our students (Atik and Yalçin, 2011; Tay et al., 2018). The cost, long waiting lists, or lack of information about this assistance all reduce the accessibility of these services to younger individuals (Anderson et al., 2017). Therefore, if the barriers associated with seeking help are too high, online self-help systems that encourage individuals to be actors in their own health can be suggested (Davies et al., 2014; Kauer et al., 2014). These programs can further include access to professional support, effective stress detection and intervention, and an opportunity to have peer support (Chan et al., 2016). This peer support, also emphasized by our female students, reduces the loneliness and isolation associated with the challenges that they can face in their academic life (John et al., 2018). Universities can encourage peer support through the development of digital communities that provide both a social resource for students in this academic environment and effective mentoring and supervision systems (Shalaby and Agyapong, 2020).

### 5.2. The needs of female students to reduce the perception of academic stress

Th female students in our study emphasized the need to obtain information correctly from the university, because this feeling of not having the necessary elements to understand the academic expectations contributes to the perception of academic stress (Maymon and Hall, 2021). Furthermore, the daily exposure of the female students to multiple sources of information brings this need to have centralized academic information and resources to reduce some of their mental load (Stich et al., 2018). The ubiquity of information can lead to confusion or even a loss of relevance of the received information that can lead the female students to feel weary regarding this inaccurate and dispersed information (Barrois et al., 2015). The university contributes to the reduction of this mental load by providing students with a learning platform that offers centralized academic resources (Chan et al., 2016) and communication spaces that are more appropriate to their age group, thus facilitating the transmission of information (Vivares and Napieralski, 2020). However, the affinities that female students have for social networks, such as Facebook or Instagram, have modulated their communication expectations (Waycott et al., 2010) and have also led them to turn away from university emails (Ha et al., 2018). Female students want communication that is more straightforward and contains more targeted and specific information, written in simple, clear language and requiring them to act (Sutton, 2017), like the communication present on social networks (Sheeran and Cummings, 2018). Yet, these social platforms are not specifically designated for academic sharing (Harith et al., 2022) and they do not provide a way to distinguish the educational context from the students' personal life (Waycott et al., 2010). This finding highlights the need for universities to promote and mentor the use of centralized information platforms for students dedicated exclusively to the academic context (Harith et al., 2022). Additionally, at the end of the focus groups, the students emphasized the benefit and value of having speaking spaces similar to what the framework of this research offers, in order to share their experiences in a supportive atmosphere that can promote the search for solutions adapted to the situation and the learning of better coping strategies (Winzer et al., 2018). However, as described in the literature, these services, already implemented by the University of Mons, are unused by our female students (Berrewaerts and Desseilles, 2016). Although the perception of these services is positive, they are also considered inaccessible or insufficient to meet their needs, or are addressed to a population with severe disorders, which limits their use by our female students (Remskar et al., 2022). Furthermore, the location of these services within the university may also be a barrier to their use for some students (Dunley and Papadopoulos, 2019; Priestley et al., 2021). For them, the university is not a sufficiently neutral place to deal with their personal difficulties. The confidentiality of the use of these services by female students can be compromised at any time by their peers or the people who are part of the university institution (Gulliver et al., 2010; Ebert et al., 2019). Therefore, in order to relieve this constraint, some female students prefer to seek psychological help outside of the university to obtain the help they seek in a setting in which they feel more confident. Nevertheless, the relocation of this help received may reduce its effectiveness in the context of good academic stress management, since the university context makes it easier to understand the students' difficulties (Storrie et al., 2010).

Therefore, how can we help these female students, especially when some of them express a need to keep their academic experience unchanged? Does this need reflect the students' ability to adapt to this environment at the expense of their stressful manifestations or does it highlight a mismatch between the resources offered and their personal interests? More research is needed with students to better understand this need and adjust action methods (Di Fabio, 2011).

### 5.3. Digital and biofeedback technologies in academic stress management: the perceptions of the female students

This last theme identifies the necessary characteristics that a potential stress management device would need to have in order to meet the students' needs. The type of technology envisioned would use biofeedback to manage stress and thus provide better detection and understanding by the students of their stress experiences through a combination of their psychological and physiological information (Schlatter et al., 2021). The female students consulted point out that a wearable device involving sensors linked to digital apps or platforms can actually increase their autonomy and would facilitate data collection in real time and in real-world settings (Yu et al., 2018a). Moreover, these devices can offer them resources that can provide them with adequate and personalized coping strategies that can improve their quality of life (Liang et al., 2020).

Furthermore, many stress detection sensors can be connected to digital platforms that can provide them with centralized academic information and stress management through different resources that will help to reduce their stress (Liang et al., 2020). They will therefore have the opportunity to have immediate and easily accessible help, anonymity, and an opportunity to connect and share experiences with their peers. This can lead to a sense of control in this academic or mental health help seeking pathway (Lupton, 2021).

Regarding stress management through their environment, as noted by our participants, new interactive technologies allow biofeedback to be better integrated into everyday environments through improved accessibility, ease of use, and comfort (Yu et al., 2018b). In smart homes in particular, stress management programs using the biofeedback method can be offered to the individual through a combination of the contextual information collected and their physiological data. Smart home equipment can thus provide relaxation programs through images, music, lighting, or relaxing smells to reduce stress (Sykianaki et al., 2019). If smart home technology equipment is not accessible to them, other wearables can also be used. Thus, using an object that can be integrated into their everyday environment, female students can create this environment of wellbeing in the room of their choice. Through a combined approach of chromotherapy, aromatherapy, light therapy and music therapy a state of wellbeing (Chappaz et al., 2017) necessary for the good mental health of female students (Roulston et al., 2018) can be promoted. However, particular attention must be paid to the conditions of adherence and acceptance of these tools by this population (Kip and van Gemert-Pijnen, 2018; Montagni et al., 2020). For our female students, the non-adherence to these tools could be due to the need to not change anything in their situation, but also to a fear of being systematically confronted with stress, whether positive or negative. This legitimate fear requires devices to ensure proper discrimination of different sources of stress to stop the spiral of pathological stress (De Witte et al., 2019). This finding also illustrates the importance of developing the content and functionality of these devices based on the characteristics of female students (Lattie et al., 2019). Thus, a co-design approach to these tools with students should be emphasized to better target their needs and increase their adherence to the developed stress management programs (Amanvermez et al., 2021). Furthermore, to maintain user engagement and to produce this change that would lead to better management of their stress, the interface of these devices must be visually attractive and easy to use, organized, and free of ambiguous features (Oyebode et al., 2020; Alhasani et al., 2022).

### 5.4. Strengths and limitations of this study

The management of academic stress through technology is a very understudied topic in the francophone scientific literature. This study is a first phase of a project that aims to offer to students a technological stress management device that comes from the academic environment. Thus, thanks to this research, we were able to discern the perceptions of a sample of female students regarding this future way of managing their stress.

From our results, we can make the following recommendations that will need to have a solid theoretical and experimental support before being integrated into technological devices for academic stress management in order to provide adequate help to female students:

° Promote centralized academic and mental health information tools to students through platforms;
° Target mental health care offerings to female students based on their characteristics;
° Enable real-time monitoring of stress levels through biofeedback sensors, preferably in a wearable form;
° Offer stress management programs in every stress detection device to allow for a reduction of the stress symptomatology;
° Provide students with an easy experience of using these tools by ensuring the relevance of the interface to increase user adherence;
° Develop and frame peer-to-peer support in these tools;
° Propose professional help from health professionals.

These recommendations need to be tested and future research can extend these suggestions by examining the different variables involved in perceptions of academic stress that will help students to develop more appropriate coping mechanisms. The goal will be to incorporate these into future technological devices that will decrease the impact of stress on students. It is first and foremost a matter of prevention and the implementation of adapted and targeted support strategies. These devices are not intended to be universal or to replace resources already available to students at universities (Harith et al., 2022). Furthermore, these tools also have limitations, including security and data protection (Wong et al., 2020). Thus, recommendations for the involvement of technology in stress management remain a suggested addition to the range of interventions available to reduce academic stress.

It should be noted that the use of biofeedback as a stress detection mechanism was not fully explored in our study. Initially, our intention was to propose a tool using biofeedback to detect stress in students. However, due to unexpected circumstances in the development of the tool, we chose to explore students' perceptions of using biofeedback to manage their stress.

Additionally, the results obtained should be interpreted with caution as they have a limit of generalizability due to the use of Braun and Clarke's (2006) method of data analysis and an exclusively female sample. In the literature, many studies show that women generally have higher levels of depression, psychological distress, and affectivity or reactivity to stressful events (Mazé and Verlhiac, 2013). Thus, our findings are specific to the female gender and cannot be generalized to the general student population. In addition, one-third of the participants are from a medical background, which may increase representations of distress as well as personal and emotional vulnerability to stress.

Indeed, these students are faced with high achievement requirements particularly due to entrance exams that condition access to the rest of their medical training, to which is added recurrent exposure to human suffering that we also find in psychology training (Dyrbye et al., 2019). In addition, our study took place during the COVID-19 health crisis that led to a lot of uncertainty in the student population and also high levels of anxiety and stress (Al-Maskari et al., 2022). This factor may also explain the high perception of stress among some of our students and the different difficulties encountered during their academic experience as they had to adapt to a new mode of distance learning and find new way of working (Haikalis et al., 2022).

### 5.5. Future directions

The use of the PSS-10 provided an objective measure of students' general stress levels, in the absence of an adequate scale that specifically measures academic stress. Future research should focus on validating an academic stress scale in French in order to adequately measure academic stress among these students. Also, to further investigate these results, a concrete prototype should be tested with other students in the future. It was found that asking students to imagine what a stress management device might look like is complicated because it requires knowledge of the topic to participate in this type of exercise.

It is also important to continue research on the use of biofeedback as a stress management tool for students. Although our study was not able to explore this dimension in depth, it provides a foundation for future research in this area. Future studies could focus on exploring the effectiveness of biofeedback as a stress management tool, taking into account students' preferences and perceptions. A better understanding of the use of biofeedback in the student context could help improve stress management strategies and promote student wellbeing.

Furthermore, in order to make this device complete, it is necessary to clarify the different stress management programs that could be offered to these students through mixed-methodology (qualitative and quantitative) data collection and data analysis studies. Subsequently, the involvement of a larger panel may be relevant as this type of device is intended for a large number of people. This consideration leads to a review of the tools and methodology currently used so that they can be adapted to this type of group.

### 5.6. Practical implications

Academic organizations can also support the management of student stress through web-based stress management programs that include the detection and awareness of stress and its effects. Until technological tools become available for students, universities, in collaboration with their psychology departments, can strengthen the adequacy of their support services by paying particular attention to the student's academic experience in the development of their interventions to reduce student stress. Additionally, in order to provide students with good resources to deal with academic stress, it is important to work with students beforehand on the source of the stress and the emotional difficulties that it may cause. This can be done through different programs that can be given in groups (e.g., psycho-educational workshops, meditation workshops, discussion groups) or in individual sessions. In addition, these programs promote the use of more productive and effective coping strategies that will help to reduce academic stress.

## 6. Conclusion

Through our qualitative research, our intentions were to understand the experience of our female university students in the management of their academic stress and then to investigate, in light of their discourse and the theoretical literature, the relevance of the involvement of technology in the management of their stress. Based on our results, we find that the different digital and biofeedback technologies can indeed be included in the management of academic stress and respond to this demand. These devices would complement the interventions already available and facilitate access to care for a larger number of students. Moreover, they can provide students with effective tools in the management of academic stress, whose specific characteristics remain to be explored in future research. Our recommendations therefore offer insights into these innovations and provide a glimpse into the expectations that female students may have.

## Data availability statement

The raw data supporting the conclusions of this article will be made available by the authors, without undue reservation.

## Ethics statement

The research protocol of this study was approved by the Ethical Committee of the University of Mons. The participants provided their written informed consent to participate in this study.

## Author contributions

M-PL contributed to the conception and design of the study. M-PL and JD organized the database and performed the qualitative analysis. M-PL and LB wrote the first draft of the manuscript and all sections of the manuscript. LB, JG, and JD contributed to manuscript revision, read, and approved the submitted version. All authors contributed to the article and approved the submitted version.

## Footnotes

^1^An electrocardiogram (ECG) is a recording of the electrical activity of the heart.

^2^An electroencephalogram (EEG) is a recording of the electrical signals of the brain.

^3^A photoplethysmogram (PPG) is used to observe blood volume variations in a tissue.

^4^An electromyogram (EMG) is a recording of electrical signals generated as a result of muscle movements.

^5^Data consulted on the official website of the Wallonia Brussels Federation: https://statistiques.cfwb.be/transversal-et-intersectoriel/genre/age-des-etudiants-de-lenseignement-superieur/.
